# Supplementary material for: Gene-Mutation-Based Algorithm for Prediction of Treatment Response in Colorectal Cancer Patients
Source: Cancers (Basel). 2022 Apr 18;14(8):2045. doi: 10.3390/cancers14082045 (PMC9030299; doi:10.3390/cancers14082045)
Supplement: Supplementary file 1 [file cancers-14-02045-s001.zip › cancers-1672156-supplementary.pdf]

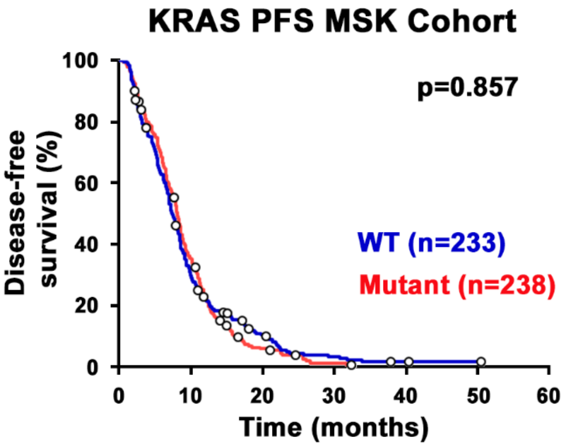

Figure S1. KRAS PFS MSK Cohort.

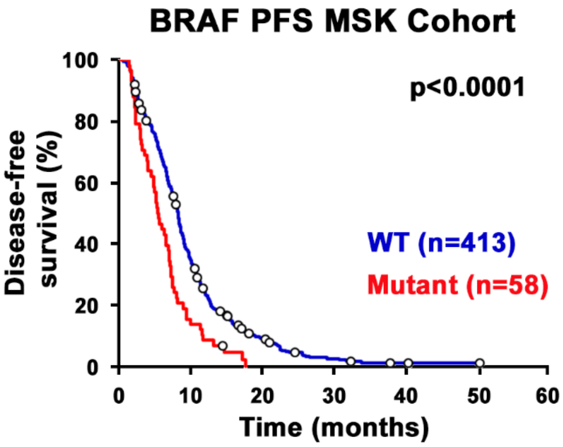

Figure S2. BRAF PFS MSK Cohort.

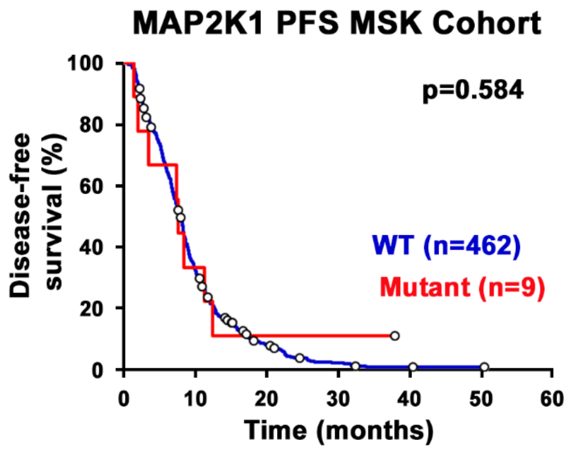

Figure S3. MAP2K1 PFS MSK Cohort.

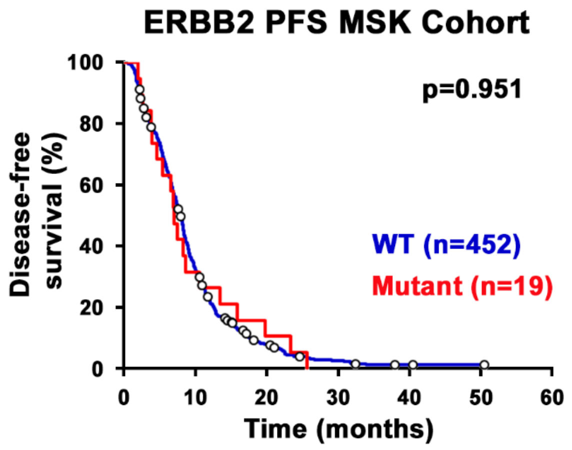

Figure S4. ERBB2 PFS MSK Cohort.

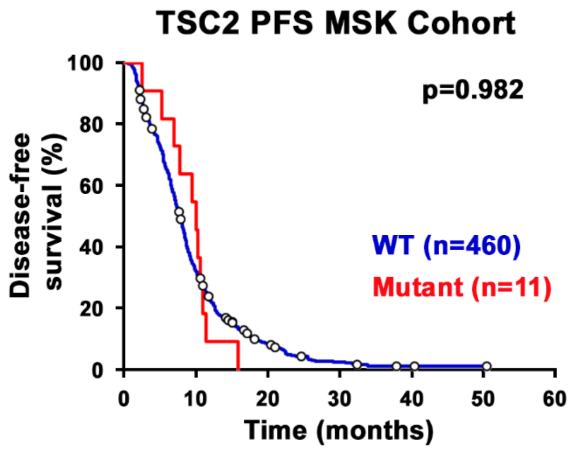

Figure S5. TSC2 PFS MSK Cohort.

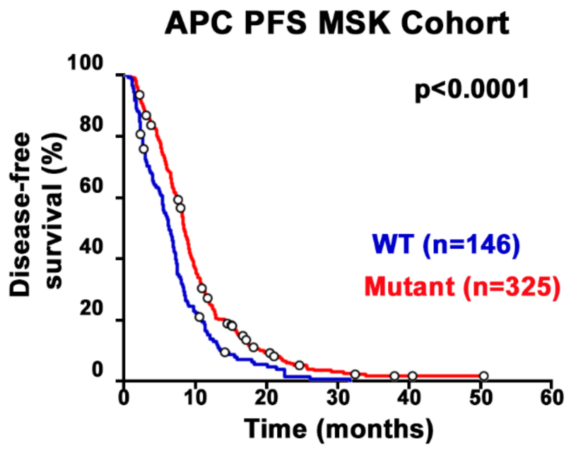

Figure S6. APC PFS MSK Cohort.

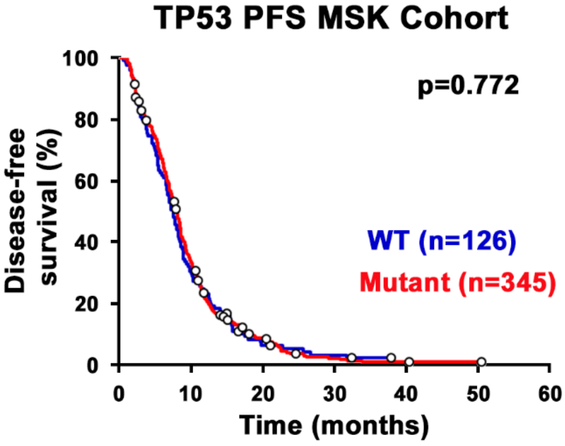

Figure S7. TP53 PFS MSK Cohort.

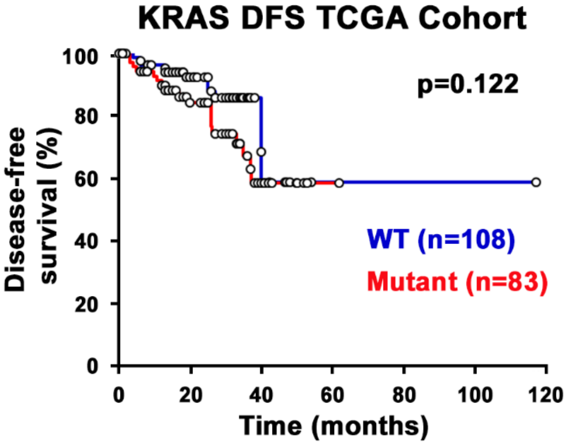

Figure S8. KRAS DFS TCGA Cohort.

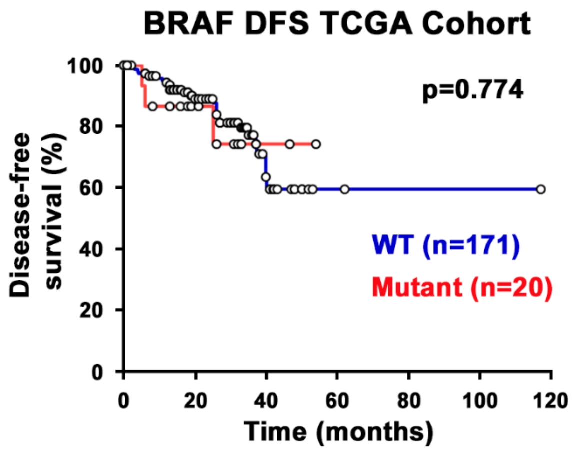

Figure S9. BRAF DFS TCGA Cohort.

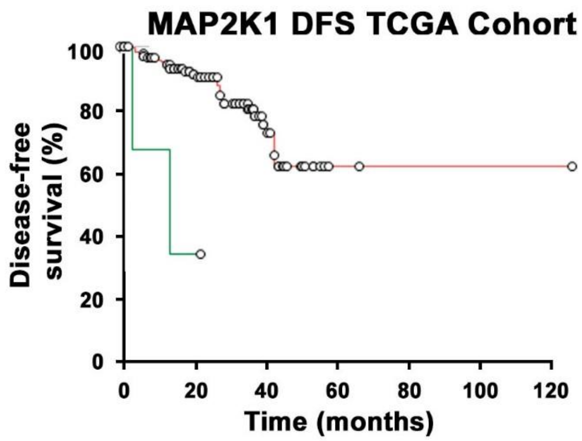

Figure S10. MAP2K1 DFS TCGA Cohort.

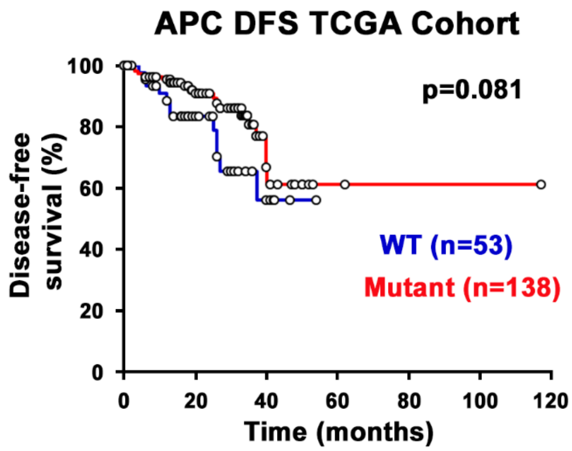

Figure S11. APC DFS TCGA Cohort.
